# Crustacean larval factor shares structural characteristics with the insect-specific follicle cell protein

**DOI:** 10.1038/s41598-019-39173-7

**Published:** 2019-02-26

**Authors:** Tomer Ventura, Chan Nguyen, Quinn P. Fitzgibbon, Tomer Abramov, Gregory G. Smith, Abigail Elizur

**Affiliations:** 1 GeneCology Research Centre, School of Science and Engineering, University of the Sunshine Coast, 4 Locked Bag, Maroochydore, Queensland 4558 Australia; 2 Institute for Marine and Antarctic Studies, University of Tasmania, Private Bag 49, Hobart, Tasmania 7001 Australia

## Abstract

Literature on the cuticle formation in larval stages of the diverse group of decapod crustaceans is lacking, as opposed to a wealth of knowledge in several insect groups. Here we provide the first glimpse of the cuticular organisation in larvae of the eastern spiny lobster *Sagmariasus verreauxi*. A bioinformatic approach applied to *S*. *verreauxi* transcriptome through metamorphosis identified for the first time a small secreted protein with multiple isoforms that is highly expressed in crustacean larvae. This protein, named crustacean larval factor (Clf) shares structural characteristics with insect follicle cell protein 3 (FCP3), an insect-specific, rapidly evolving protein, with spatial-temporal regulated expression that is restricted to follicular cells during the production of the vitellin coat. Furthermore, we identified the FCP3 domain in additional structural proteins in multiple arthropod groups. Recombinant Clf inhibited *in vitro* calcium carbonate crystalline precipitation, in keeping with the finding that the spiny lobster larval cuticle is mainly composed of amorphous calcium carbonate. In addition, the recombinant Clf was shown to bind chitosan. Taken together, this research identifies two novel structural domains with lineage-specific expansion across arthropods. In crustaceans, Clf is found predominantly in larvae and the spatial-temporal regulated FCP3 factor occurs as a domain identified in multiple structural proteins across arthropods. Given the shared ten cysteines backbone between the Clf and FCP domains, a shared evolution is suggested and should be further explored.

## Introduction

Arthropods are defined by their epidermis which produces a segmented, jointed, and hardened chitinous exoskeleton^1^. The cuticular composition has been thoroughly studied in quite a few decapod crustacean species including crabs^2–4^, and clawed lobsters^5^. The crustacean cuticle is mainly composed of layers of chitin scaffolds linked by binding proteins which also sequester minerals, predominantly crystalline (calcite, aragonite and vaterite) and amorphous calcium carbonate^6^, and to a lesser extent, calcium phosphate^7^. It was shown that as many as 50% of the cuticle-associated proteins have chitin-binding domains^8^, of which some were also shown to include regions with high content of negatively-charged amino acids as well as phosphorylated amino acids which provide electrostatic force to sequester and stabilise calcium carbonate at its amorphous state^9–11^. Interestingly, there is no reference to the cuticular protein composition of larval life stages (prior to metamorphosis) in decapod crustaceans. This gap in knowledge is in great contrast with the highly defined larval cuticular composition of several crustacean-derived insects^12^.

Spiny lobsters oceanic life begins with an abbreviated nauplius stage. Within hours to days from hatching, the nauplii transition into a leaf-shaped flat and transparent larvae (phyllosoma) – an exceptionally prolonged oceanic phase that incrementally accumulates the nutritional load required to transition into the benthic life stages. Following 11 to 24 consecutive molts (depending on the species), over a period of up to two years, the phyllosoma reach the point of energy saturation required for metamorphosis into an intermediary free-swimming (nektonic) phase called a puerulus. Unlike the predatory phyllosoma, the puerulus is a non-feeding phase, utilizing the energy stored as phyllosoma to fuel its hundreds of miles journey to the benthic habitat. While resembling the adult body plan it is transparent and gradually accumulates pigments. When reaching the benthic habitat, following an unknown signal, the puerulus molts into a juvenile completing the transition from a pelagic, oceanic stage to the benthic, juvenile development^13^.

The transparency of the spiny lobster cuticle during the oceanic (phyllosoma) and nektonic (puerulus) phases prior to metamorphosis into the benthic juvenile stage, requires highly-structured organization of the cuticle. The phyllosoma cuticle serves also as the key respiratory organ, enabling gas exchange without gills, which develop later, in the puerulus stage. The ventral epidermal layer of the phyllosoma cephalic shield was found to be thicker and enriched with mitochondria compared with the dorsal epidermal layer, indicating the ventral side is the one in charge of gas exchange in the phyllosoma^14^. In barnacles, a specialized epithelial layer with a potential dual role in osmoregulation and biomineralization was identified^15^, portraying a clear pattern of specialized epithelia that enable respiration and ion exchange across mineralized cuticles in distantly related crustacean taxa. It was suggested that increased temperatures and decreased dissolved oxygen in marine environments might significantly affect the vulnerable spiny lobster larvae due to their immature respiratory system^16^. Better understanding of the molecular mechanisms which facilitate the gas/ion exchange capacity across mineralized cuticles might prove useful in developing bio-inspired materials and will also contribute to our understanding of the potential consequences climate change holds for species with confounding respiratory capacity.

A high proportion of the eastern spiny lobster *Sagmariasus verreauxi* transcriptome (25%) was found to significantly change in expression throughout metamorphosis from the oceanic phyllosoma to the nektonic puerulus larval stage^17^. This massive change in expression, which occurs within a few days, can hold the key for better understanding the mechanism which governs metamorphosis and indeed several gaps in crustacean endocrinology were resolved by addressing this transcriptomic resource^17–20^. Metamorphosis in spiny lobsters is bi-phasic; instead of one molt event which transforms the pelagic life form to the benthic life form, an intermediate stage is present in the form of the nektonic puerulus stage. This gradual and prolonged metamorphosis^17^ enables sampling individuals throughout metamorphosis at greater resolution than that available in any other crustacean taxon. The puerulus is a transitional non-feeding stage which transfers the oceanic phyllosoma to the benthic habitat, where it will complete its metamorphosis to the juvenile benthic form. While there are significant anatomical changes which accompany the puerulus-juvenile transition, the external morphology of this transition is subtle. The puerulus looks externally like a miniature transparent version of the juvenile and while swimming to the benthic habitat it gradually expresses pigmentation. When reaching the benthic habitat, it molts to become a benthic juvenile. The latter change does not seem dramatic as compared to the earlier change between the oceanic phyllosoma and the puerulus stage, since the phyllosoma is a transparent, flattened larva with little resemblance to the later life stages of spiny lobsters.

The present study uses a bioinformatic approach applied to the transcriptome through metamorphosis of *S*. *verreauxi*^17^ to identify for the first time a small secreted protein with multiple isoforms that is highly expressed in crustacean larvae in this research. This protein, named ‘crustacean larval factor’ shares structural characteristics with insect follicle cell protein 3 (FCP3), an insect-specific, rapidly evolving protein, with spatial-temporal regulated expression that is restricted to follicular cells during the production of the vitellin coat^21^. Furthermore, the current research identified the FCP3 domain in additional structural proteins in other arthropod groups. Recombinant crustacean larval factor protein inhibited *in vitro* calcium carbonate crystalline precipitation and bound chitosan, in keeping with the finding that the spiny lobster larval cuticle is mainly composed of amorphous calcium carbonate.

Taken together, this research identifies two novel structural domains with lineage-specific expansion within arthropods. Crustaceans Clf is found predominantly in larvae and the spatial-temporal regulated FCP3 factor is found to be a conserved domain across arthropods in many structural proteins.

## Materials and Methods

### Scanning electron microscopy

For scanning electron microscopy (SEM), samples that were snap frozen in liquid nitrogen and stored in −80 °C were transferred into 100% Ethanol for 4 h at room temperature. Samples were then air-dried for 10 min and then mounted onto stubs with carbon tape for thin layer platinum sputter coating (Hitachi ion sputtering apparatus, E5000) for 1 min. The specimens were examined by a Hitachi S-2500 SEM at 10–15 kV.

### Bioinformatics analysis

The transcriptome of whole individuals sampled from five developmental stages throughout metamorphosis of *S*. *verreauxi* (in duplicates, including six phyllosoma and 4 puerulus^17^), was screened for unknown sequences (no hit with e-value < 1.00 × 10^−5^ in any of the five databases previously tested^17^), with a fold-change ≥10 between the five sub-stages with *P* value ≤ 0.05 (with FDR; according to RPKMs as previously calculated^17^). The total 386 contigs that were filtered using these parameters were retrieved from the database using a python script (https://github.com/IdoBar/get_from_fasta_gui.git) and the resulting sequences were converted to amino acids (aa) of the most probable open reading frame (ORF) using OrfPredictor (proteomics.ysu.edu/tools/OrfPredictor.html).

The predicted ORFs were submitted to SignalP 4.1 (http://www.cbs.dtu.dk/services/SignalP/) and two sequences with a signal peptide (above the D-cutoff for SignalP-noTM networks of 0.45 and D-cutoff for SignalP-TM networks of 0.50) were retrieved, followed by BLASTp against nr, swissprot and tsa_nr databases at NCBI (http://www.ncbi.nlm.nih.gov). The sequences were also used for BLASTp or tBLASTn against transcriptomes available for decapod crustaceans, including the blackback land crab *Gecarcinus lateralis* Y-organ, the Australian redclaw crayfish *Cherax quadricarinatus*, the giant freshwater prawn *Macrobrachium rosenbergii*, the white shrimp *Litopenaeus vannamei* and back against the Eastern spiny lobster transcriptome^17^. The BLAST was done iteratively with each subsequent hit that was identified against all of the above-mentioned databases, enabling broader identification of similar sequences.

Once this methodology did not retrieve new results, the entire list was trimmed to the domain with the conserved 10 cysteine residues and these trimmed sequences were BLASTed against NCBI nr, nt and TSA databases and swissprot database. Multiple sequence alignments with ClustalW alignment^22^, followed by Neighbor-Joining method phylogenetic analysis^23^ were performed using MEGA6^24^. Putative disulfide bridges were calculated using DISULFIND^25^. Disulfide bridges with confidence level of 6–9 (on a scale of 0–9, where 0 is unlikely to occur and 9 is very likely to occur) were considered valid. Using predicted disulfide bridges, a contact file was generated to guide three-dimensional (3D) modelling in I-TASSER (http://zhanglab.ccmb.med.umich.edu/I-TASSER/).

### Expression analysis

Expression analysis to validate RPKM values that were previously calculated^17,26–28^ was performed using RT-PCR as previously described^26^ with slight modifications. In brief, *S*. *verreauxi* samples were obtained from cultured animals supplied by Institute for Marine and Antarctic Studies, University of Tasmania, Hobart. Lobsters were reared as previously described^29,30^. Total RNA was isolated from whole phyllosoma and puerulus individuals using Trizol® Reagent (Invitrogen), according to manufacturer’s instructions, followed by reverse transcriptase reaction containing 1 μg total RNA extracted from each individual, using Tetro cDNA Synthesis Kit (Bioline, Australia) following manufacturer’s instructions. The cDNA was then amplified by PCR using F1 polymerase (Fisher Biotec, Australia) using 1 μL cDNA as a template, 100 nM of forward (F) and reverse (R) specific primers under the following conditions: 94 °C for 3 min, followed by 30 cycles of 94 °C for 30 s, annealing at 60 °C for 30 s, elongation at 72 °C for 45 s, followed by a final extension at 72 °C for 10 min. Primers were designed using Primer 3 (http://bioinfo.ut.ee/primer3-0.4.0/) and produced by Sigma–Aldrich. Amplicons were then electrophoresed on a 1.5% agarose gel stained with ethidium bromide and visualized under UV light.

### Recombinant protein production

Recombinant protein was produced in the methylotrophic yeast *Pichia pastoris*, as previously described^31^. The pPIC9K vector was used to drive expression of Sv-Clf1. A 6 X His tag was inserted at the N’ terminus of the protein encoding sequence, with the signal peptide truncated.

### *In vitro* calcium carbonate precipitation assay

*In vitro* precipitation of calcium carbonate was achieved by mixing equal volumes (100 μl) of 20 mM CaCl_2_ and 20 mM Na_2_CO_3_ solutions, a short vortex, spin down and incubation for 4 hours at room temperature with constant shaking at 200 rpm. Prior to the vortex, 20 μl of either water, Sv-Clf1, or human insulin (200 ng/μl), were added to the solution. Following incubation, the precipitate was pelleted by mild centrifugation at 1,500 × g for 5 min, followed by removal of the liquid above. The precipitate was then spread over a glass cover slip, air-dried, and mounted onto stubs with carbon tape for observation by SEM as described above.

### Chitosan binding assay

Chitosan (CS), a deacetylated form of chitin (97.5% deacetylation) was prepared at 0.025% (w/v) in 1% acetic acid and pre-heated to 55 °C for 30 min. 50 µl of Sv-Clf1 (200 ng/µl) was added to an equal volume of either CS 0.025% or 1% acetic acid, and vortexed immediately at 13,000 g for 1 min, followed by incubation at RT overnight. The tubes were the spun down at 10,000 g for 5 min at 4 °C then the supernatant, which contained the unbound protein, was deglycosylated with PNgase F (NEB) following the manufacturer’s instructions. The treated protein was then electrophoresed on a 16% SDS-PAGE Tricine gel and stained with Coomassie blue.

## Results

**Figure 1:**
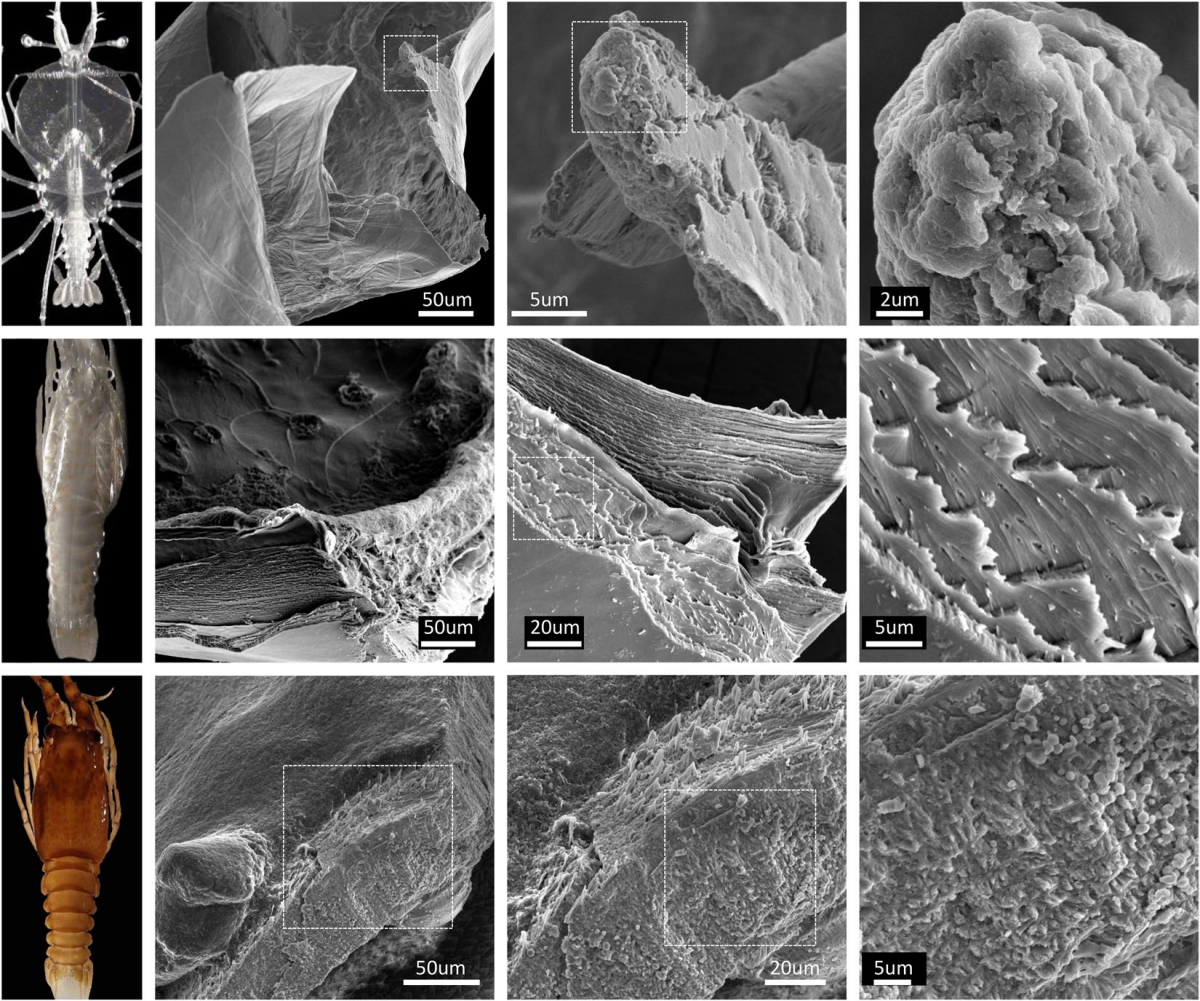
Ultrastructure of the eastern spiny lobster cuticle across metamorphosis. Left panels show an intermolt stage of last phyllosoma (instar 17; top), early post molt puerulus (mid) and intermolt juvenile of the eastern spiny lobster *Sagmariasus verreauxi*. The cuticle of the corresponding stages was observed and photographed under scanning electron microscopy with increasing magnifications from left to right. Dashed rectangles signify the region zoomed in on the photo to the right.

Comparison of the cuticle in phyllosoma (Fig. 1, top panels) with puerulus (mid panels) and juvenile (bottom panels), shows that the phyllosoma has a very thin cuticle with amorphous calcium carbonated tightly packed in a spherule shape (Fig. 1, top-right panel) which makes it difficult to observe the layers of chitin. It is less than 10 μm thick, as compared with at least 5 times thicker cuticle in the puerulus and juvenile stages. It is shown that in the clear puerulus stage, soon after molt, the chitin layers are devoid of mineral and there are canals that run longitudinally (Fig. 1, mid-right panel). At the juvenile stage the chitin layers are stacked with mineral, resembling cuticles defined in other decapods at post-metamorphic stages, with less distinction between regions of the cuticle (i.e. epicuticle, exocuticle and endocuticle).

**Figure 2:**
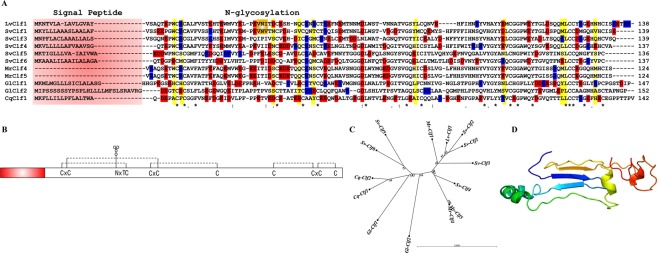
Key features of the Clf domain. (**A**) Multiple sequence alignment of representative Clfs across five decapod species. Only sequences with complete or near-complete sequence were chosen to represent the key features including a variable signal peptide (highlighted in light red), 10 conserved cysteine residues (highlighted in yellow) and a high negative (E, D; highlighted in red) to positive (K, R; highlighted in blue) amino acids ratio. N-glycosylation motif (highlighted in orange) is found in the most highly expressed Clf identified in *Sagmariasus verreauxi* (SvClf1) and *Litopenaeus vannamei* (LvClf1). Other represented species include *Macrobrachium rosenbergii* (Mr), *Gecarcinus lateralis* (Gl) and *Cherax quadricarinatus* (Cq). Asterisk marks fully conserved identical amino acids, colon marks strongly similar conserved amino acids and full stop marks weakly similar conserved amino acids. (**B**) A linear model of the conserved structure with signal peptide at the N’-terminus (red), a conserved N-glycosylation motif (NxT) and 10 conserved cysteine residues that are predicted to stabilize a three-dimensional structure through 5 disulphide bridges (dashed lines). Additionally, there is a high abundance of negatively charged residues, and two conserved tyrosine residues which could be phosphorylated (A). (**C**) A radial phylogenetic tree of the aligned Clf amino acid sequences, showing dispersed phylogeny pattern of *S*. *verreauxi* Clfs. (**D**) A putative three-dimensional structure produced in I-TASSER, considering the predicted disulphide bridges.

**Figure 3:**
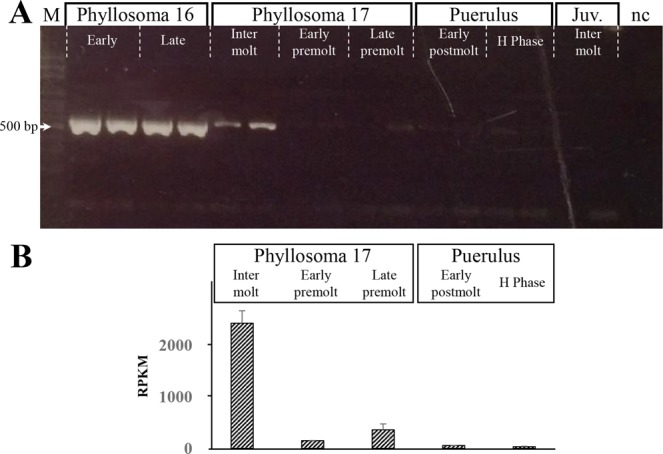
Expression pattern of spiny lobster Sv-Clf1 encoding transcript. Temporal expression of *Sv-Clf1* across metamorphosis in *S*. *verreauxi* was tested using RT-PCR (**A**) as well as calculated *in silico* as RPKM (**B**). In both cases there is a clear validation of the phyllosoma stage-specific expression of *Sv-Clf1*.

Analysis of the transcriptomic changes during the phyllosoma-puerulus transition highlighted a high proportion of genes putatively encoding unknown proteins. Screening these unknown proteins for characteristics such as signal peptides, PFAM domains and structural motifs highlighted several transcripts which encode a conserved sequence characterized by a signal peptide, ~100–200 aa in total length, with 10 conserved cysteine residues that are predicted to stabilize a putative three-dimensional structure by forming 5 disulphide bridges (Fig. 2). The transcript with highest expression was termed *S*. *verreauxi* crustacean larval factor 1 (*Sv-Clf1*), due to its high expression specifically in larvae. Five additional isoforms were identified and assigned similar names with different numbers (i.e. *Sv-Clf2*, *3*). Digital gene expression of *Sv-Clf1* followed by RT-PCR showed it has extremely high expression in phyllosoma 16, relatively high expression in phyllosoma 17 and decrease in expression to basal levels in puerulus. Expression was not detected in juveniles as well as in adult tissues (Fig. 3).

Using BLASTP and tBLASTn of these sequences against other publicly available crustacean databases, more candidate Clfs were identified (Fig. 2). These included 9 isoforms in the giant freshwater prawn *M*. *rosenbergii* (assembled in house using SRA files downloaded from NCBI BioProject PRJDB3040), where once again, expression was calculated digitally to be very high in larvae but absent in post larvae. A highly similar sequence was identified in the closely-related Pacific white shrimp *L*. *vannamei* in a transcriptome derived from early life stages^32^. In the transcriptome of the Y-organ of the black land crab *G*. *lateralis*^33^, two isoforms were identified and three were identified in a transcriptome of adult tissues from the Australian red claw crayfish *Cherax quadricarinatus* (assembled in house using SRA files downloaded from NCBI BioProject PRJEB5112). All complete putative ORFs were aligned (Fig. 2A), showing high conservation of the cysteine residues, which suggest a conserved structural configuration is stabilized via 5 disulphide bridges. The predicted ORFs encoded by the most abundantly expressed *Clfs* in *S*. *verreauxi*; Sv-Clf1 and Sv-Clf2 share also a putative N-glycosylation with *L*. *vannamei* Lv-Clf1 (Fig. 2B). The Clf ORFs alignment served to construct a radial phylogram, showing various isoforms are scattered regardless of the species they stem from (Fig. 2C). The predicted three-dimensional structure of Sv-Clf1 following cleavage of the putative signal peptide and formation of 5 disulphide bridges (predicted using disulfind), support an assumptive structural feature for this protein (Fig. 2D). The sequences of all Clfs presented in this study are given in Supplementary File 1.

**Figure 4:**
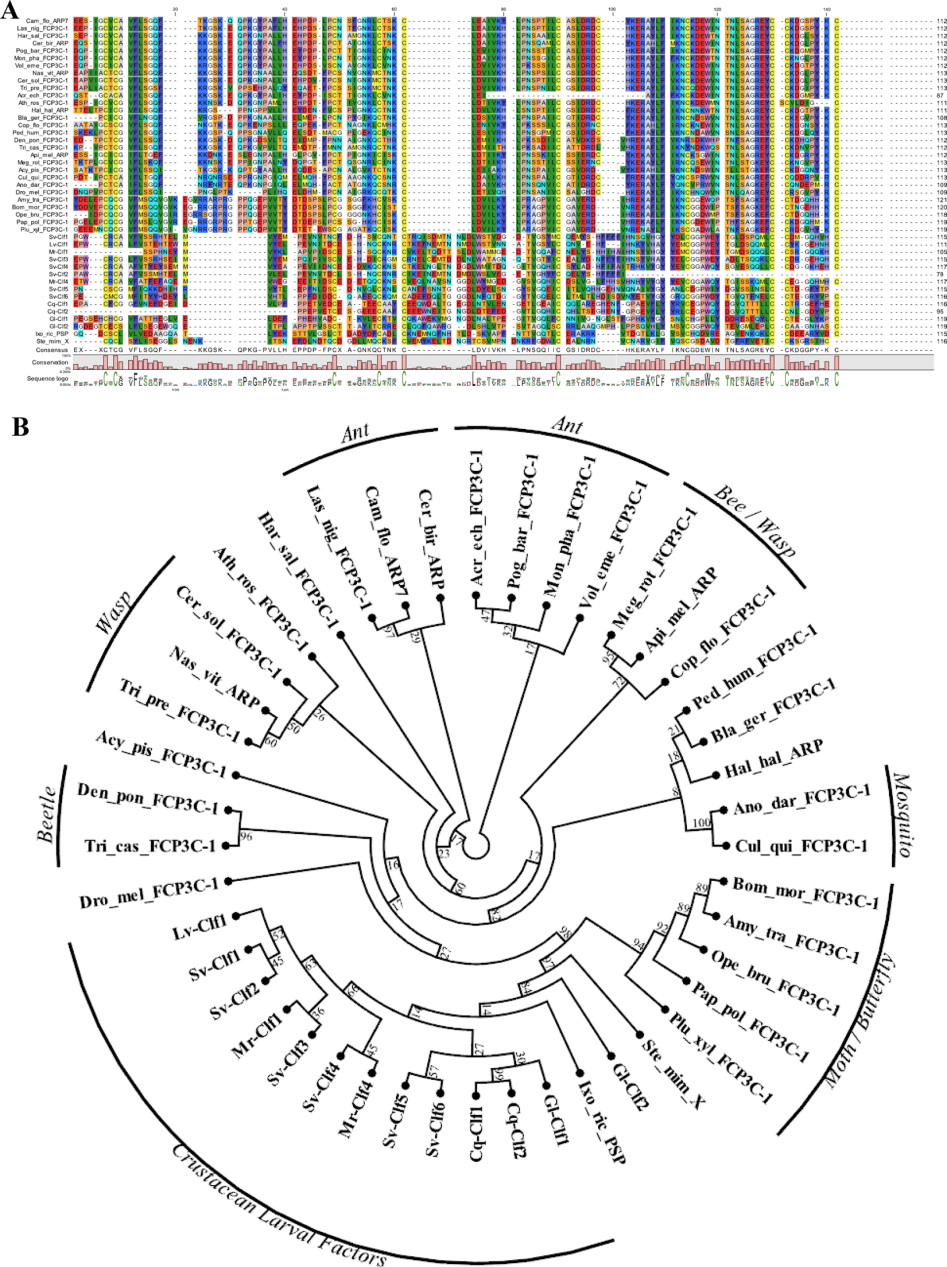
Evolutionary relationships of Clf and FCP3 domains. (**A**) Multiple sequence alignment of Clf (bottom) and FCP3 (top) domains in arthropods. The evolutionary relationship is suggested based on the 10 conserved cysteine residues. With that, it is notable that there are two regions with considerably large gaps between the FCP3 domain and the Clf1 domain and the fact that while in Clf there is a high abundance of negatively charged residues, in FCP3 there is a high abundance of positively charges residues. (**B**) A radial cladogram of Clf and FCP3 domains. Clfs cluster with two chelicerate putative Clf-like proteins as well as lepidopteran FCP3 proteins. Species and proteins abbreviations are elaborated in Supplementary File 1.

Using BLASTP and tBLASTn against publicly available databases (NCBI nr and nt, as well as swissprot) of all organisms no similar sequence was identified with the exception of several arthropod sequences which included the follicle cell protein 3C1 (FCP3C1), and Armadillo repeat containing proteins (where the region of similarity is at the C terminus of the protein which was not previously defined as a domain or motif) and two putative secreted proteins from a tick and a spider (Supplementary File 1). Multiple sequence alignment, followed by tree construction (Fig. 4) showed that the cysteines residues are aligned with a distinct gap between the Clfs and the FCP3 and Armadillo derived sequences, although the tick and spider sequences showed closer homology with the Clfs than with the insect derived FCP3 and Armadillo proteins (Fig. 4).

**Figure 5:**
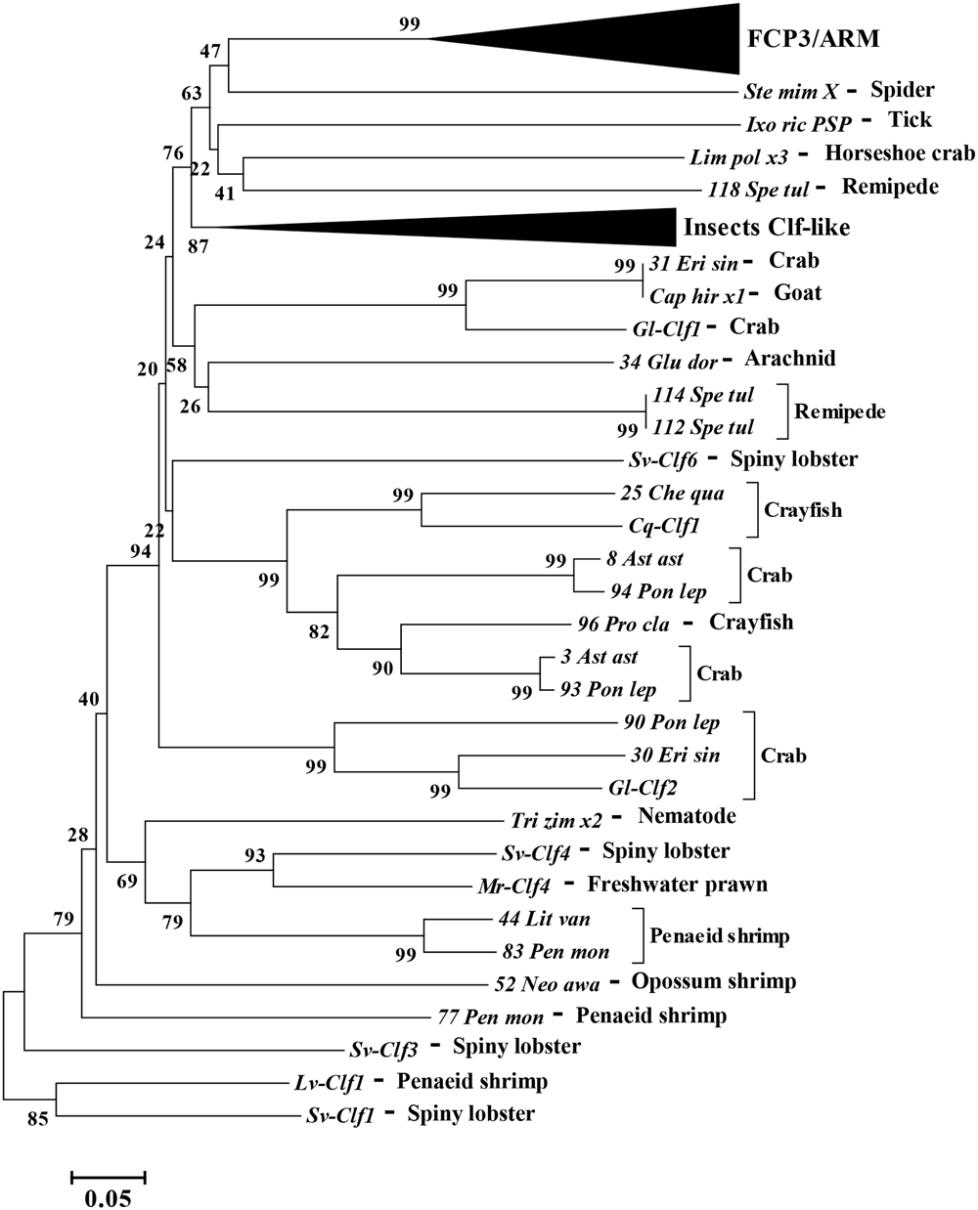
Evolutionary relationships of Clf domain. The evolutionary history was inferred using the Neighbor-Joining method^23^. The optimal tree with the sum of branch length = 11.94477201 is shown. The confidence probability (multiplied by 100) that the interior branch length is greater than 0, as estimated using the bootstrap test (1000 replicates is shown next to the branches^35,36^). The tree is drawn to scale, with branch lengths in the same units as those of the evolutionary distances used to infer the phylogenetic tree. The evolutionary distances were computed using the p-distance method and are in the units of the number of amino acid differences per site. The analysis involved 71 amino acid sequences. All positions containing gaps and missing data were eliminated. There were a total of 52 positions in the final dataset. Evolutionary analyses were conducted in MEGA6^24^.

tBLASTn of Sv-Clf1 against the NCBI TSA databases of arthropods generated 276 results, of which 226 were from crustaceans. These 276 resulting aligned sequences were shortlisted to 127 based on coverage (at least 75% coverage with Sv-Clf1) and removal of identical sequences, to eliminate redundancy (Supplementary File 1). No significant similarity was found when same search was applied for poriferans, cnidarians, nematodes, annelids, molluscs, echinoderms and hemichordates. Intriguingly, one sequence had a perfect match with Sv-Clf1 cysteine arrangement, found in the chordates databases (Supplementary File 1). When BLASTed against NCBI nr database, this sequence gave similarity with two sequences from a nematode, 3 sequences from a horseshoe crab and one from a lancelet, all predicted or hypothetical, yet all with the conserved cysteines (Supplementary File 1). Analysis of the Amino Acid Composition (in MEGA6) showed there are no residues other than Cysteine with a conserved abundance. The phylogenetic relationship between Clfs, FCP3s and additional structural proteins is given in Fig. 5.

**Figure 6:**
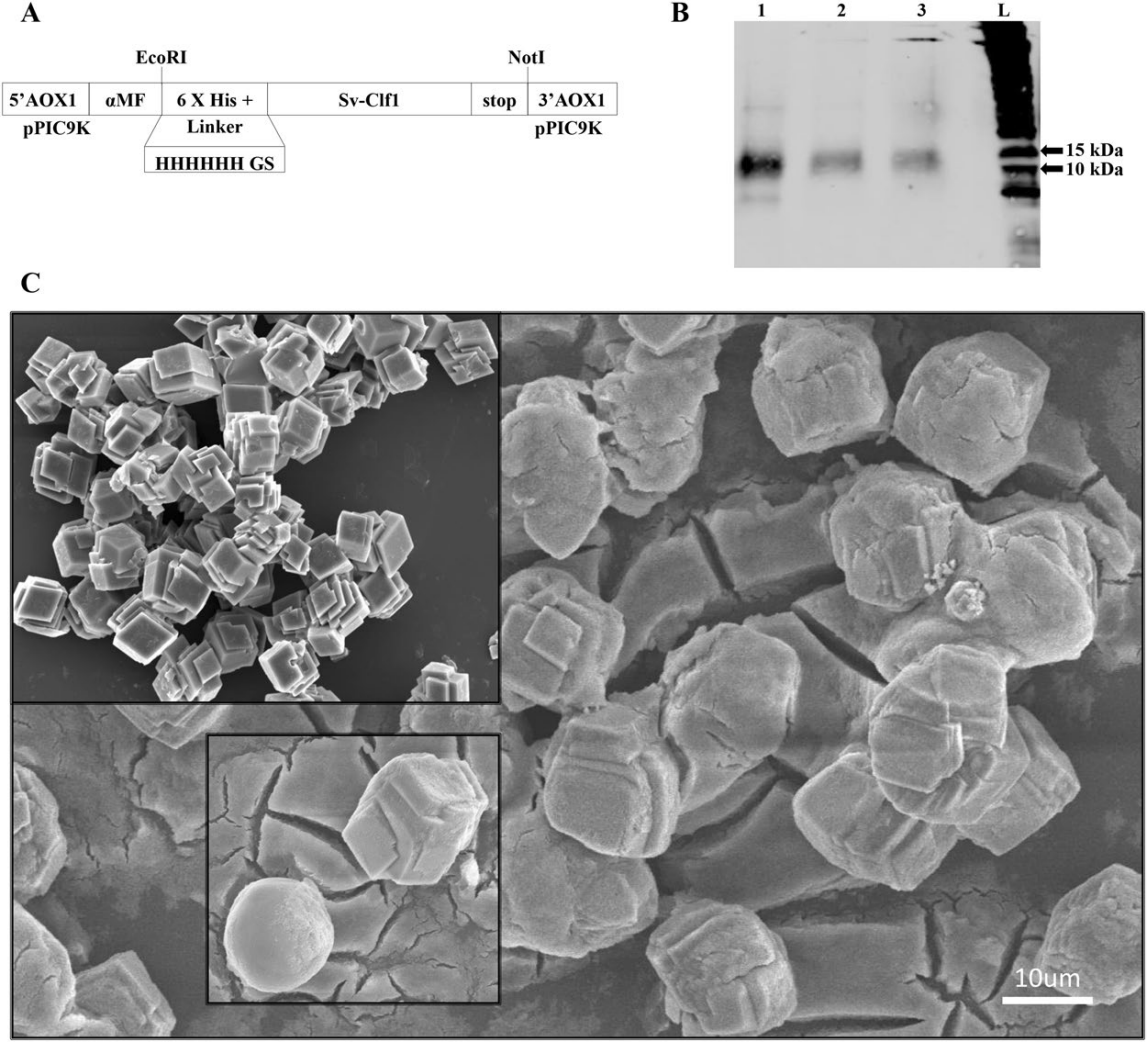
Inhibition of calcium carbonate crystallization *in vitro* by incubation with recombinant Sv-Clf1. (**A**) Linear schematics of the vector design for producing the recombinant Sv-Clf1 in *Pichia pastoris*. The sequence encoding the ORF of Sv-Clf1 was inserted upstream the alpha mating factor, between EcoRI and NotI restriction sites on the pPIC9K vector, with a 6XHis tag at the N’-terminus. (**B**) Following geneticin resistance selection, three colonies were isolated and grown in small scale. The extracted protein was tested in a western blot analysis using an Anti-His antibody, showing all three colonies (1–3; L = molecular weight ladder) produced the expected size recombinant protein, with the highest yield in colony 1 which was produced in 1 L for the Calcium Carbonate precipitation assay. (**C**) Calcium Carbonate precipitation in the absence of protein and in the presence of a recombinant human insulin gives the same result of tightly packed calcite (top left inset). In the presence of the recombinant Sv-Clf1 the Calcium Carbonate precipitates in an amorphous shape, with larger crystals which occasionally form spherules (bottom right inset).

Recombinant Sv-Clf1 produced in *P*. *pastoris* using the expression vector pPIC9K (Fig. 6A) showed the expected size band of 14.8 kDa (Fig. 6B). The best expressing colony served for protein production in 1 L which yielded ~600 µg, in keeping with previous production of other proteins using this system^31^. Human insulin, or negative control with no protein gave similar results when incubated with calcium chloride and sodium carbonate. Following incubation, a calcite precipitate was formed (Fig. 6C, top left inset). When incubated with recombinant Sv-Clf1 on the other hand, a less dense precipitate formed (Fig. 6C, background) and in some cases a spherical shape was observable (Fig. 6C, bottom left inset).

**Figure 7:**
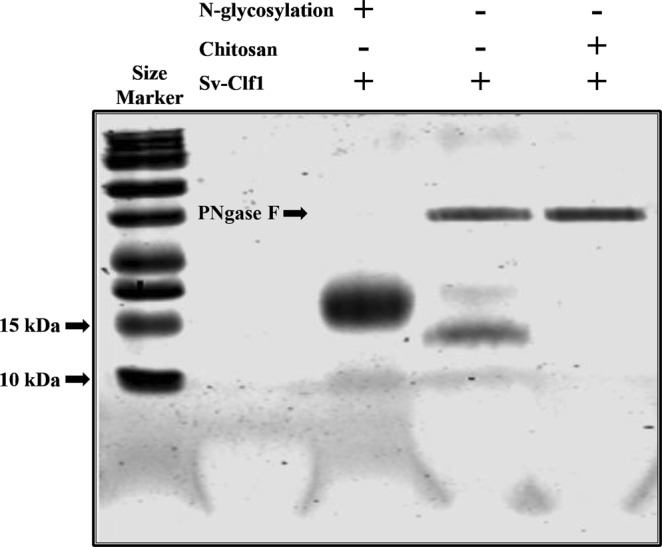
Recombinant Sv-Clf1 N-glycosylation and chitosan binding capacity. 16% SDS-PAGE Tricine gel stained with Coomassie blue shows that the recombinant Sv-Clf1 is glycosylated due to the brighter, smeary and higher band when not treated with PNgase F. When treated with PNgase F a basnd of the expected size (~14.8 kDa) appears, in keeping with the western blot result. When pre-incubated with chitosan and precipitated, the lack of Sv-Clf1 indicates it co-precipitated with the chitosan.

Sv-Clf1shares a conserved N-glycosylation site with Sv-Clf2 as well as Lv-Clf1 (Fig. 2A,B). Coomassie staining of the recombinant Sv-Clf1 with and without glycosylation shows that without glycosylation (following PNgase F treatment) Sv-Clf1 runs at approximately 14.8 KDa (in keeping with the western blot result; Fig. 6B), while with glycosylation the size and intensity of Sv-Clf1 are higher (Fig. 7). Chitosan binding assay shows that the recombinant Sv-Clf1 binds chitosan (Fig. 7).

## Discussion

This study addresses a gap in knowledge with regards to the protein composition of decapod crustacean larval cuticle. The research is based on the transcriptomic library of the eastern spiny lobster *S*. *verreauxi* which showed a high proportion of the transcriptome is changing within days in the frame of the metamorphic transition^17^. This spiny lobster metamorphosis is uniquely suitable for this research, given the prolonged duration, large transparent larvae and the ability to trace the molt stage towards metamorphosis as it occurs^17^. A first glimpse into the composition of the phyllosoma cuticle as compared with puerulus and juvenile cuticle is provided (Fig. 1), showing that the phyllosoma has a unique spherule-like arrangement of amorphous calcium carbonate. In the early postmolt puerulus stage a clear plywood structure of the chitin layers is observed, in keeping with former literature of decapod cuticle in later stages^2,6^. In the intermolt juvenile stage, this chitinous scaffold is sequestering minerals. Further research is required to analyse the mineral composition at each stage.

Using a bioinformatic approach to filter larval specific factors with signal peptides, this research reveals a new structural domain defined here as ‘crustacean larval factor’ (Clf), since it is predominantly expressed in the larval stages in crustaceans. As many as nine different isoforms could be identified in the giant freshwater prawn *M*. *rosenbergii*, all retain a high expression level, specifically in the larval stages. In multiple decapod species, several Clfs were identified. The main characteristics of Clfs are signal peptide, in keeping with their function in cuticle formation, followed by a pattern of 10 highly-conserved cysteine residues which most likely give rise to 5 disulphide bridges that stabilise a cysteine-knot three-dimensional structure. Interestingly, other conserved features of the Clfs identified include abundance of negatively-charges amino acids as well as two tyrosine residues. Given that negatively-charged amino acids as well as phosphorylated amino acids were shown to sequester amorphous calcium carbonate and inhibit its precipitation as calcite^9–11^, the expression of Clfs in the larvae, where the cuticle is composed of amorphous calcium carbonate raises the possibility that this is a conserved domain that supports amorphous calcium carbonate precipitation in crustacean larval cuticles.

When searching outside Crustacea, a highly similar sequence was identified in two chelicerate species: the castor bean tick *Ixodes ricinus*, and the velvet spider *Stegodyphus mimosarum* (Fig. 4). Given that insects are land-adapted derived crustaceans^34^, a thorough search was performed in insects although only somewhat similar sequences were identifiable. These were predominantly the FCP3 members which were found in *D*. *melanogaster* to express specifically in the follicular cells when the vitellin coat is produced^21^. Unlike Clfs, the FCP3 proteins share a net positive charge and while they show a conserved arrangement of the 10 cysteine residues after the signal peptide, two distinct gaps set these sequences apart (Fig. 4). Further examining the FCP3 proteins, it was found that these are in fact yet another highly abundant structural domain present in many insect species, either as a secreted protein like in the case of FCP3 itself, or nuclear proteins that include an armadillo domain at the N’-terminus and the FCP3 domain at the C’-terminus. The evolutionary link between the Clfs and FCP3 domain containing proteins was further examined using a phylogenetic tree analysis (Figs 4 and 5). In both cases there are multiple isoforms in each species, suggesting these are rapidly evolving proteins. Their conserved, highly regulated spatial-temporal expression patterns throughout Arthropoda indicates they play significant roles in development although the phylogenetic analysis cannot conclusively define them as two gene families which have originated from a common ancestor. Given the high prevalence of FCP3 proteins in insects and high prevalence of Clfs in crustaceans, in light of the pancrustacean theory^34^, further research in *Myriapoda* could resolve this issue.

When specifically addressing the putative function of the Clfs, a recombinant protein of the most abundantly expressed Clf from *S*. *verreauxi* (Sv-Clf1) was shown to inhibit the precipitation of calcite *in vitro* (Fig. 6), perhaps due to the net negative charge and abundance of putative phosphorylation sites (Fig. 2). Sv-Clf1 was also found to bind chitosan (Fig. 7), suggesting it plays a dual role in both binding the chitin/chitosan scaffold and sequestering minerals in an amorphous shape. There is little information about the role of FCP3 and here we show that it is in fact a domain present in multiple structural proteins across arthropods. The parallel evolution of the two spatial-temporal regulated Clf and FCP3 structural domains, both with 10 conserved cysteine residues predicted to provide a backbone for the 3-dimensional structure, suggest they might share a common ancestral gene that has rapidly evolved across the arthropod lineage.

## Supplementary information

Supplementary File 1

## Data Availability

All novel domains identified in this study are presented in full in Supplementary File 1.
